# Hydrophobic Surface Array Structure Based on Laser-Induced Graphene for Deicing and Anti-Icing Applications

**DOI:** 10.3390/mi15020285

**Published:** 2024-02-17

**Authors:** Mian Zhong, Shichen Li, Yao Zou, Hongyun Fan, Yong Jiang, Chao Qiu, Jinling Luo, Liang Yang

**Affiliations:** 1 Institute of Electronic and Electrical Engineering, Civil Aviation Flight University of China, Deyang 618307, China18731838513@163.com (H.F.); 2 School of Mathematics and Physics, Southwest University of Science and Technology, Mianyang 621010, China; y_jiang@swust.edu.cn; 3 College of Aviation Engineering, Civil Aviation Flight University of China, Deyang 618307, China; chaoqiu1987@163.com; 4 Chongqing Institute of Green and Intelligent Technology, Chinese Academy of Sciences, Chongqing 400714, China; luojinling@cigit.ac.cn

**Keywords:** laser-induced graphene, periodic linear array, hydrophobic, electric heating deicing, anti-icing application

## Abstract

The exceptional performance of graphene has driven the advancement of its preparation techniques and applications. Laser-induced graphene (LIG), as a novel graphene preparation technique, has been applied in various fields. Graphene periodic structures created by the LIG technique exhibit superhydrophobic characteristics and can be used for deicing and anti-icing applications, which are significantly influenced by the laser parameters. The laser surface treatment process was simulated by a finite element software analysis (COMSOL Multiphysics) to optimize the scanning parameter range, and the linear array surface structure was subsequently fabricated by the LIG technique. The generation of graphene was confirmed by Raman spectroscopy and energy-dispersive X-ray spectroscopy. The periodic linear array structure was observed by scanning electron microscopy (SEM) and confocal laser imaging (CLSM). In addition, CLSM testings, contact angle measurements, and delayed icing experiments were systematically performed to investigate the effect of scanning speed on surface hydrophobicity. The results show that high-quality and uniform graphene can be achieved using the laser scanning speed of 125 mm/s. The periodic linear array structures can obviously increase the contact angle and suppress delayed icing. Furthermore, these structures have the enhanced ability of the electric heating deicing, which can reach 100 °C and 240 °C within 15 s and within 60 s under the DC voltage power supply ranging from 3 to 7 V, respectively. These results indicate that the LIG technique can be developed to provide an efficient, economical, and convenient approach for preparing graphene and that the hydrophobic surface array structure based on LIG has considerable potential for deicing and anti-icing applications.

## 1. Introduction

Graphene has a remarkable single-layer, two-dimensional (2D) honeycomb lattice structure interconnected by sp^2^ hybridization. Currently, graphene is the thinnest and strongest substance available. Owing to its ultrahigh carrier migration rate (105 cm^2^/V·s), excellent mechanical properties (1 TPa), large theoretical specific surface area (2630 m^2^/g), extraordinary thermal conductivity (5 kW/m·K), and commendable chemical stability [1,2,3], graphene stands out among other materials. Various methods, such as the pyrolysis repeated stripping [4], vapor deposition [5], reduced graphene oxidization [6], and SiC epitaxial growth [7] methods, have been employed to prepare graphene. However, these conventional preparation methods suffer from issues such as low efficiency, complicated operation, uncontrollable quality, and strict and harsh conditions. A breakthrough occurred in 2014 when Jame et al. discovered the use of laser direct writing technology to directly obtain graphene on polyimide (PI) films [8]. Consequently, owing to its straightforward process, wide range of substrate options and lasers available for use, high-precision control capabilities, and several other advantages, laser-induced graphene (LIG) technology has emerged with great potential for efficient large-scale graphene preparation while promoting low-C and environmentally friendly development. 

The LIG technology uses laser surface treatment to induce photothermal or photochemical reactions on the material surface [9], thereby breaking the chemical bonds between C atoms and other elements in the substrate material and facilitating their recombination into graphene structures. Several scholars have employed this technology for realizing high-performance capacitors [10], batteries [11], sensors [12], water decomposition [13], optical components [14], photothermal conversion devices [15], thermal brakes [16], and medical applications [17]. Owing to their small spot diameters, lasers possess a unique advantage in constructing intricate structures. As a combination of laser and graphene manufacturing, the LIG technology offers lighter multifunctional hydrophobic surfaces compared to traditional silicone rubber or fluorosilane-based superhydrophobic surfaces [18,19,20,21]. Moreover, it is more environmentally friendly and has greater potential for cost-effectiveness. Currently, this technology has advantages in preparing graphene-based superhydrophobic surfaces and expanding their application to areas such as anticoronavirus methods based on hydrophobic and Joule thermal properties [22]. In addition, the LIG technology leverages high-precision patterning and distinctive electrowetting characteristics to achieve directional water transport function [23]. Furthermore, micro/nanostructures can be constructed to enhance the microfluidic mixing effect [24]. Based on the structure, conductivity, and wettability of LIG, biosensing and energy storage devices can be fabricated for multiplex open microfluidic environments [25]. However, various factors such as laser parameters (e.g., laser power [26], spot size [27], scanning speed [28]), substrate materials (e.g., PDMS [24], PI paper [29]), surface doping [30], and processing patterns (e.g., microcolumns [31], periodic linear arrays [32,33], grids [24]) have different degrees of influence on the LIG preparation process. The exploration of the inherent characteristics of LIG and further development of its application value are essential for sustainable development. Therefore, employing the finite element software to simulate laser surface treatment and establishing the simulation results as the foundation for preparation and design represents an economically and environmentally effective approach [34].

Herein, LIG preparation was investigated using finite element software analysis. A multiphysics coupling model of the commonly used PI film under laser surface treatment was established using COMSOL Multiphysics software. The classical periodic linear array structure was used as the processing pattern. The effect of laser scanning speed on the surface heat accumulation and thermal stress in laser-induced PI films was analyzed and discussed. Based on the simulation analysis results, experimental preparation was performed, which verified the consistency between actual observations and simulated outcomes. Moreover, particular emphasis was placed on studying the effects of different laser scanning speeds on the surface morphology and quality of graphene. The prepared periodic linear array graphene exhibited variations in surface roughness, water contact angle (WCA), hydrophobicity, and delayed icing performance under various scanning speeds. In addition, the application of Joule thermal expansion revealed the broad potential application of the LIG technology in microstructure array construction, hydrophobic anti-icing, and electric deicing.

## 2. Experimental Process

### 2.1. Materials

**Figure 1:**
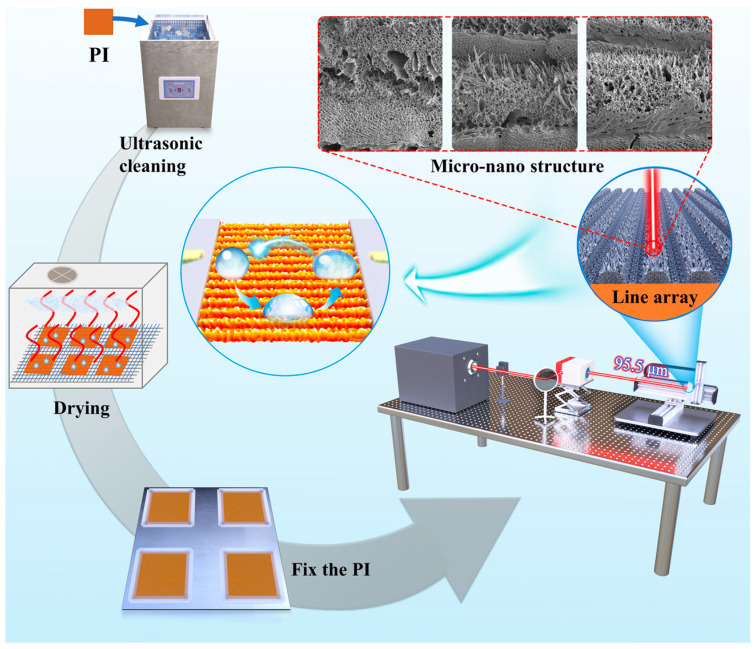
Preparation flow chart of LIG hydrophobic surface.

In this experiment, a 125 µm thick PI film (DuPont Kapton HN, China, purchased from Alibaba Mall) measuring 150 mm × 150 mm was utilized as the substrate for laser processing. Laser surface treatment was performed using a 10.6 µm continuous CO_2_ laser (SYNRAD P-150, Synrad, Inc., Bedford, MA, USA) at a 20 kHz frequency. Before laser processing, an ultrasonic cleaning machine (CR-010S, Shenzhen Chunlin Cleaning Equipment Co., Ltd., Shenzhen, China) using distilled water and an air blast thermostat (DHG-202, Shaoxing Subo Instrument Co, Ltd., Shaoxing, China) were employed for sample cleaning (30 min) and drying (40 min), respectively. Laser output power was measured and calibrated using an optical power meter (BIM-7614-0250F, Hangzhou Boyuan Optoelectronics Technology Co., Ltd., Hangzhou, China). Subsequently, a laser control system comprising a vibroscope and parameter setting software called Marking Mate was used for film load adjustment and defocusing of the laser spot on a 3-axis mobile platform. The preparation process is shown in Figure 1.

### 2.2. Characterization Equipment

A focused ion beam scanning electron microscope (FIB-SEM, Thermo Scientific Helios 5 CX, Waltham, MA, USA) was used to observe surface micromorphology and perform surface scanning energy-dispersive X-ray spectroscopy (EDS). The degrees of surface carbonization and structural defects were measured using a laser confocal Raman spectrometer (Renishaw inVia, New Mills, UK) with a laser light source at 532 nm wavelength. The microscopic morphology of a 1415 µm × 1061 µm surface area was observed via three-dimensional (3D) confocal laser scanning microscopy (CLSM, Keyence VK-X1000, Higashi-Nakajima, Japan). A contact angle tester (Kruss DSA100, Mainz, Germany) and the accompanying ADVANCE interface analysis software were used to observe the state of water droplets on the graphene surface. The surface resistance of graphene was measured using a four-probe tester (RTS-8, Guangzhou four-probe technology instrument Co., Ltd., Guangzhou, China), and the average value of nine randomly measured points was considered as the resistance value. The electric Joule thermal properties of the graphene surface were stabilized using a linear DC power supply (RIGOL DP700, Beijing, China), and the surface heating infrared images were collected using an infrared thermal imager (Fluke TiX650, Everett, WA, USA). Industrial optical lens electron microscopy (TDR 48MP FHD Camera V8, China) was employed to observe the freezing state of water droplets on the graphene surface.

### 2.3. Simulation Parameters

First, we developed a physical model of a PI substrate with dimensions of 1.5 cm × 1.5 cm × 125 µm (L × D × H) using the geometry module of COMSOL Multiphysics software based on the laser processing pattern size (1 cm × 1 cm) and PI film thickness. To simulate the laser action, we modeled the laser beam as a Gaussian distribution on the XY interface and expressed the light intensity distribution on each horizontal interface along the optical path as follows [35]:

(1) $$I(r)=I_{0}e^{-2r^{2}/\omega^{2}}=\frac{2P}{{\pi R}^{2}}e^{-2r^{2}/R^{2}},$$

where *P* represents the laser power, *r* represents the distance from any point in the spot plane to the spot center, and *R* represents the radius of the laser to the spot in the plane.

According to Equation (1), a moving heat source was established by combining triangular wave and piecewise functions to simulate the effect of actual laser processing on the film surface. The temperature field on positive and negative film substrate surfaces and the temperature-induced thermal stress and temperature and stress changes during scanning at the positive/back spot of films were monitored and recorded using a boundary probe. A solid heat transfer physical field was designed to facilitate the absorption, diffusion, and radiation of temperature from the moving heat source [35,36]. The thermal diffusion conduction for a material is expressed as follows:

(2) $$\rho C_{p}\frac{\partial T}{\partial t}+\rho C_{p}\upsilon \cdot \nabla T+\nabla \cdot (-k\nabla T)=Q+Q_{T},$$

where *ρ* represents the material density, *C_p_* represents the constant pressure heat capacity of the material, *T* represents the temperature, *v* represents the velocity vector, *k* represents the thermal conductivity of the material, *Q* represents the heat absorbed by the material, and *Q_T_* represents the thermoelastic damping of the material.

The conversion of laser energy absorbed by a film into heat is determined by the absorption and reflection of light on its surface during laser action; this is described as follows:

(3) $$-n\cdot q=I_{0}\cdot \theta ,$$

where *I*_0_ represents the laser energy and *θ* represents the light energy conversion efficiency of the material (*θ* = 0.1778) [37].

A simulation was performed to analyze the thermal stress process occurring on the PI thin film surface. The back of the PI thin film was fixed as a constraint while the remaining surface was free. The calculation formula for thermal strain is expressed as follows:

(4) $$\varepsilon_{th}=\alpha (T)(T-T_{ref}),$$

where *ε_th_* represents thermal strain, *α* represents the coefficient of thermal expansion, *T* represents the film surface temperature, and *T_ref_* represents the volume reference temperature.

The relevant parameters of the PI thin film were set in accordance with the Kapton HN series data books and related literature [29,38].

## 3. Results and Discussion

### 3.1. Simulation Analysis and Characterizations

**Figure 2:**
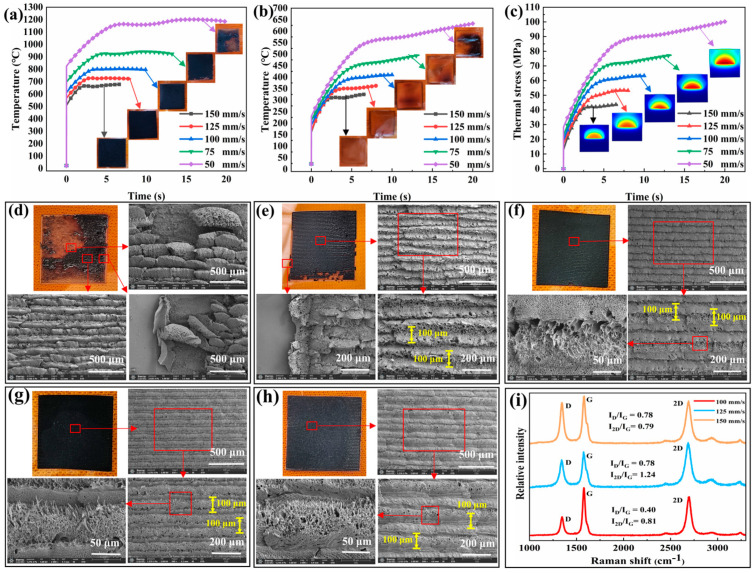
(**a**–**c**) Finite element simulation results of the PI surface directly written using a CO_2_ laser between temperature trend and surface treatment time: positive temperature, negative temperature, and heat stress. The illustrations are as follows: (**a**) front picture of sample; (**b**) backside view of sample; (**c**) simulated stress distribution map during halfway scan; (**d**–**h**) SEM images of LIG arrays at scanning speeds of 50, 75, 100, 125, and 150 mm/s, scale bar: 500 μm, 200 μm, 50 μm; (**i**) Raman spectra of the LIG array surface at scanning speeds of 100, 125, and 150 mm/s.

The simulation results of temperature and heat strain, which are obtained from the multiphysics field coupling model, are shown in Figure 2a–c. Figure 2a shows that an increase in scanning speed while keeping other laser parameters constant leads to a decrease in the steady-state temperature on the film surface. This observation agrees with the fundamental principles governing rapid heat accumulation during laser scanning. The simulation results in Figure 2c show the thermal stress exerted on the back of the PI film. The 3% yield point stress, which is ~61 MPa for the PI film, indicates that when the scanning speed is <100 mm/s, the thermal stress applied to the back of the film will exceed its threshold and result in folding. These findings agree with the actual observations depicted in Figure 2b. Furthermore, a consistent agreement exists between our finite element model and real laser effects, indicating that strategic use of our model can offer valuable insights for experimental purposes.

The microstructure of the thin film surface was observed after laser treatment. The SEM images in Figure 2d–h display the graphene surface at a scanning speed range 50–150 mm/s (with a 25 mm/s spacing interval). The linear array formed via laser scanning is distinctly visible, albeit with variations in the microstructure within the linear array. At a 50 mm/s scanning speed, several shed and defective structures can be observed on the linear array surface, with certain areas exhibiting overlap between track paths. These phenomena result from the low scanning speed, which leads to high temperature accumulation around each spot and graphene generation area, causing them to exceed their designated size and resulting in track overlap. In addition, excessive thermal stress on the film surface induces a decrease in adhesion performance for surface products, resulting in detachment. In addition, a 75 mm/s scanning speed resulted in surface product and microstructure deteriorations, which are attributed to excessive heat accumulation. However, compared with the 50 mm/s scanning speed, there is a reduction in the deterioration regions and overlap rate of linear array trajectory, indicating an improvement achieved due to the increased scanning speed. The linear array surface exhibited consistent coverage but contained a considerable amount of voids caused by gas precipitation. At the scanning speeds of 100–150 mm/s, a uniform linear array structure with gaps between tracks and submicrometer structure was observed on the surface. At the 100 mm/s scanning speed, rough burr structures filled up these gaps, whereas at 125 mm/s scanning speed, fine and medium holes and sheet burrs occupied them, forming the trajectory with decreasing density of large hole defects. At 150 mm/s scanning speed, fused PI filled up these gaps, and mesoporous and microburr structures formed the trajectory, accompanied by scattered large pore defects whose appearance is related to the thermal decomposition degree of deep-layer PI films.

**Figure 3:**
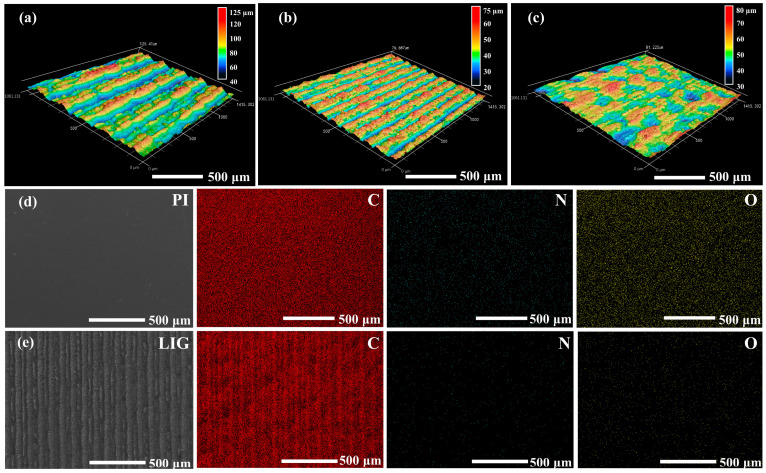
(**a**–**c**) CLSM test diagram of the graphene linear array surface at scanning speeds of 100, 125, and 150 mm/s. (**d**,**e**) EDS test diagram of the graphene linear array surface on PI film at a scanning speed of 125 mm/s, scale bar: 500 μm.

The composition and microscopic binding structure of the linear array structure were further analyzed and investigated using Raman spectroscopy, laser confocal imaging, and EDS elemental analysis on surfaces with reduced defects at scanning speeds of 100, 125, and 150 mm/s (Figure 2i and Figure 3). Raman spectra were examined to identify the characteristic peaks associated with graphene: D, G, and 2D peaks at approximately 1345.9, 1577.9, and 2692.6 cm^−1^ for the three scanning speeds. The ratios between these peaks were analyzed to explain their distinctive features. The linear array surface with a scanning speed of 100 mm/s exhibited an I_D_/I_G_ ratio of 0.4 and an I_2D_/I_G_ ratio of 0.81, where the lower I_D_/I_G_ ratio reflected a reduced presence of defect structures compared with smoother SEM images previously observed. For the linear array surface with a scanning speed of 125 mm/s, an I_D_/I_G_ ratio of 0.78 was observed along with an I_2D_/I_G_ ratio of 1.24, indicating an increased defect distribution as reflected by the higher I_D_/I_G_ ratio, the formation of multilayer or stacked graphene fragments results in an increase in the I_2D_/I_G_ ratio. Meanwhile, for the linear array surface with a scanning speed of 150 mm/s, the I_D_/I_G_ (0.78) and I_2D_/I_G_ (0.79) ratios remained relatively unchanged because of the rough burr structure present between the gaps in the linear array. The lower I_2D_/I_G_ ratio is attributed to varying degrees of carbonization in multilayer PI films due to lower scanning speed [39]. In summary, an increase in scanning speed promotes an enhancement of defect density in graphene. Moreover, appropriately increasing the scanning speed also facilitates the formation of multilayer and flake-like graphene structures. Considering anti-icing performance requirements, scanning speeds of 125 and 150 mm/s are more suitable for constructing rough surface structures. Conversely, for deicing performance requirements, a scanning speed of 100 mm/s is better suited for constructing stable electrically heated deicing structures.

The surface geometry of the graphene linear array is shown in Figure 2a–c. Decreasing the laser scanning speed increases protrusions along the linear array, while the gullies represent the gaps between laser tracks. The scanning speed of 100 mm/s generated a rectangular microrough gully structure with a consistent height of ~120 µm. At the scanning speed of 125 mm/s, evenly distributed trapezoidal gullies were observed within the linear array, exhibiting a height of ~75 µm and a protruding width of ~100 µm, which is consistent with the SEM measurements. Gradually increasing the scanning speed to 150 mm/s introduced discrete bumps along the graphene linear array surface, characterized by relatively dispersed heights ranging from 60 to 80 µm. This phenomenon is attributed to the uneven heat accumulation due to high scanning speeds.

Analyzing the SEM images, Raman spectral characteristics, and laser confocal imaging data revealed that the surface quality and uniformity for graphene generation reach their optimum at a scanning speed of 125 mm/s. To further validate the effect of the laser treatment on increasing C content while minimizing other major elements, EDS analysis was performed on the PI film and surface prepared at a scanning speed of 125 mm/s. The EDS elemental mapping images for C, N, and O are shown in Figure 3d,e. Comparing the C distribution between the two samples revealed that laser processing considerably enhanced the C content on the surface. In addition, within each scanned locus, a higher C content lies by the center spots of the laser beam because of the complete thermal decomposition due to the high energy in this region. Moreover, a noticeable reduction in N and O contents was observed on the graphene linear array surface compared with those present in the PI film. This observation agrees with the LIG formation mechanism under photothermal effects.

### 3.2. Surface Hydrophobic Properties and Delayed Icing Effect

**Figure 4:**
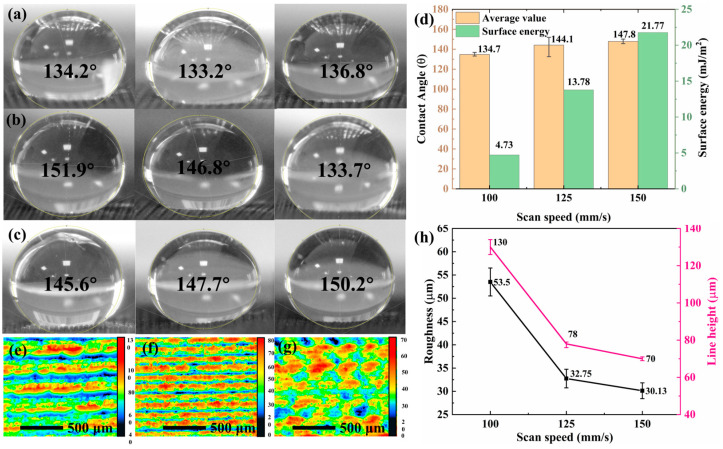
Surface hydrophobicity of graphene linear array at scanning speeds of 100, 125, 150 mm/s: (**a**–**c**) side views of the surface WCA; (**d**) surface WCA and surface energy; (**e**–**g**) 2D height maps of surfaces; (**h**) surface roughness and line height.

The linear array structure with a specific height exhibited remarkable hydrophobicity and potential. Consequently, the WCA and surface energy of the linear array surface prepared at 100, 125, and 150 mm/s scanning speeds were subjected to testing. The WCA and surface energy were randomly measured thrice, and the average values were considered as the final results. The results are shown in Figure 4a–d. A slight increase in the WCA was observed with an increase in scanning speed. Nevertheless, all samples displayed exceptional hydrophobicity (CA = 134.7°–147.8°). Notably, there was a considerable increase in the overall surface energy from 4.73 to 21.77 mJ/m^2^, which consequently enhanced the water droplet adhesion on the surface.

To further investigate the effect of laser scanning speed on contact angle and surface wetting characteristics, we performed tests on the 2D height and roughness of the graphene linear array at three scanning speeds (Figure 4e–h). The top-down structure revealed in the 2D height map demonstrated a considerable degree of consistency with features observed in the laser confocal 3D image analysis. Using the Gwyddion software for processing the 2D height map (CLSM) enabled us to obtain measurements for roughness and line height across all three surface structures. An increase in scanning speed decreased the convex height for the graphene linear array. Furthermore, SEM microscopic images indicated a reduction in surface irregularities and burr structures, resulting in a considerable decrease in roughness from 53.4 to 30.13 µm. This trend was also reflected by changes in line height. The decrease in roughness further enhanced the liquid–solid contact region, which resulted in compromised surface anti-wetting properties and increased surface energy, thereby enhancing droplet adhesion.

**Figure 5:**
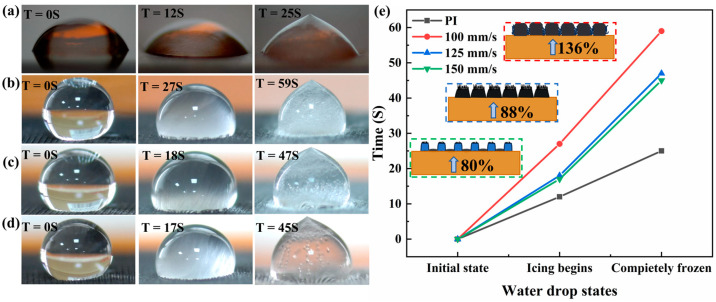
Schematic and mechanism of delayed icing effect on graphene linear array surface at low temperatures: (**a**–**d**) the corresponding time of the initial state of the droplet on the surface and the beginning of ice core and complete icing at scanning speeds of 100, 125, and 150 mm/s; (**e**) comparison of delayed icing effect and delayed icing mechanism in terms of time.

Based on the analysis of the aforementioned basic test results, the delayed icing effect of the graphene linear array surface prepared under the three scanning speeds was examined, and the results are shown in Figure 5a–d. Owing to the periodic convex and concave structure of the graphene linear array surface, it forms a gully-like topography that effectively reduces the contact region between the droplets and the surface. This structure feature facilitates air pocket formation between the droplets and surface, thereby reducing the temperature diffusion rate and serving as a heat insulation buffer during temperature fluctuations on the linear array surface. To observe this delayed icing effect, it was affixed onto a small cooling plane maintained at a stable temperature of −23 °C to minimize the heat transfer from beneath to droplets. The ice nuclei began forming on the PI film at *t* = 12 s, with complete freezing occurring at *t* = 25 s. Moreover, the tested graphene linear array surface exhibited varying degrees of delayed icing effects when subjected to scanning speeds of 100, 125, and 150 mm/s. To visually and more effectively compare these effects, time was considered as an evaluative scale with point plots representing the initial state (not ice), onset state (ice nuclei appearance), and complete icing state (Figure 5e). The surface revealed a subtle enhancement effect on the appearance of delayed ice nuclei, whereas it considerably improved the performance of the delayed complete icing of droplets. Based on the aforementioned observations on surface microstructure, a diagram depicting the delayed complete icing effect resulting from different surface morphology structures is shown in Figure 5e. When the scanning speed was set to 100 mm/s, cavitation was more likely to occur at the liquid–solid contact interface due to the presence of intricate perforations in the linear array track and burr structure within gaps. In addition, the heightened graphene elevations weakened the bond between frozen droplets and embedded microstructures on the liquid–solid contact surface, resulting in a 136% increased delay effect. At the scanning speed of 125 mm/s, burrs were formed on the array surface while smoother pores constituted gaps. Nevertheless, some degree of cavitation could still be achieved.

### 3.3. Electrothermal Properties and Deicing Effect

**Figure 6:**
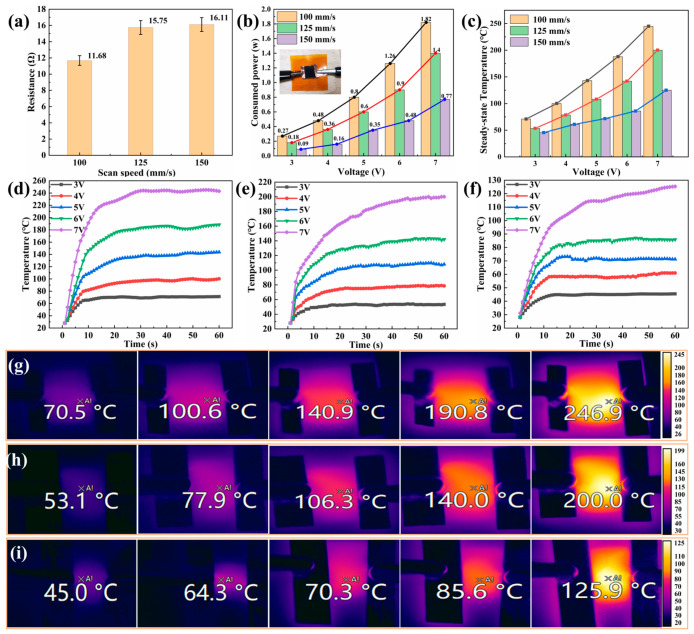
Laser-induced electrothermal properties of graphene surface at scanning speeds of 100, 125, and 150 mm/s: (**a**) square resistance of graphene linear array surface; (**b**) electrothermal connection method and power consumption of graphene surface; (**c**) electrothermal steady-state temperature at a voltage of 3–7 V; (**d**–**f**) trend chart of electric heating temperature change on the surface at different voltage values; (**g**–**i**) infrared thermal imaging map corresponding to the surface electrothermal steady-state temperature.

Owing to the presence of a C conductive region on the linear graphene array surface, tests were performed to evaluate surface resistance, power consumption, and Joule thermal performance. Surface resistance was determined using a four-probe tester to measure the block resistance at nine points and calculate an average value (Figure 6a). Lower scanning speeds facilitated a more thorough decomposition of elements within the PI film, resulting in graphene with reduced surface resistance. A Cu foil was connected at both ends of the linear array to assess the connection resistance and electric Joule thermal performance, and the results are shown in Figure 6b,c. A decrease in surface resistance increased the power consumption and electric Joule thermal performance. The power consumption rule for the graphene surface follows the electric power calculation formula *P* = *U*^2^*/R*. Notably, with a DC voltage power supply ranging from 3 to 7 V, the minimum power consumption reached 0.09 W while the maximum power consumption was only 1.82 W. Figure 6d–f show the temperature change trend of the graphene surface, highlighting its exceptional responsiveness to heat and remarkable energy conversion efficiency. Within just 15 s, the surface temperature can rapidly increase to 100 °C, and within just 60 s, it can even reach a considerably high temperature of 240 °C. As shown in Figure 6g–i, the precise temperature regulation within the range 70–240 °C can be achieved by adjusting scanning speeds. In particular, a scanning speed of 100 mm/s enabled the accurate temperature control. Similarly, scanning speeds of 125 and 150 mm/s facilitated the accurate temperature regulation within the ranges 50–200 °C and 45–125 °C, respectively. These flexible and controllable thermal adjustment capabilities and hydrophobicity are indispensable for anti-icing and electrothermal deicing technologies.

## 4. Conclusions

In this study, COMSOL Multiphysics was employed to develop a simulation model of temperature and stress coupling on a PI thin film surface under laser surface treatment. Experiments were performed with reference to the simulation results. The graphene state on the positive surface of the PI thin film and the fold on the back of the film were consistent with those predicted by the simulation model. Using SEM, the microscopic effects of laser scanning speed on the surface morphology of graphene were investigated. The observations revealed that the microstructure changed from burnt paste shedding to a uniform distribution of pores before developing into microburr shapes of pores. Raman spectroscopy tests performed on the surfaces with more regular graphene morphology at scanning speeds of 100, 125, and 150 mm/s revealed that superior-quality graphene was generated at scanning speeds of 100 and 150 mm/s. In addition, higher scanning speeds were not conducive to meeting the quality requirements of graphene generation. EDS tests were performed on the graphene linear array surface at a scanning speed of 125 mm/s, yielding excellent quality results that confirmed the reduction in N and O and other element content due to gas precipitation. Based on the findings from CLSM testing, contact angle measurements, surface energy analysis, roughness evaluation, and delayed icing experiments were systematically performed to investigate the effect of scanning speed on surface structure. Consequently, an enhanced contact angle and a suppressive effect on delayed icing were observed. Thus, this research expands upon the electric heating functionality of the graphene linear array surface because it can reach 100 °C within 15 s or even 240 °C within 60 s under a DC voltage power supply ranging from 3 to 7 V. These findings provide an effective approach for manufacturing low-cost, precision-controllable components that have hydrophobic, anti-icing, and deicing capabilities and electric heating properties.

## Data Availability

Data are contained within the article.

## References

[1] Stoller M.D., Park S., Zhu Y., Stoller M.D., Park S., Zhu Y., An J., Ruoff R.S. (2008). Graphene-based ultracapacitors. Nano Lett..

[2] Balandin A.A. (2011). Thermal properties of graphene and nanostructured carbon materials. Nat. Mater..

[3] Han T., Nag A., Simorangkir R.B.V.B., Afsarimanesh N., Liu H., Mukhopadhyay S.C., Xu Y., Zhadobov M., Sauleau R. (2019). Multifunctional flexible sensor based on laser-induced graphene. Sensors.

[4] Novoselov K.S., Geim A.K., Morozov S.V., Jiang D.E., Zhang Y., Dubonos S.V., Grigorieva I.V., Firsov A.A. (2004). Electric field effect in atomically thin carbon films supplementary. Science.

[5] Jia K., Zhang J., Zhu Y., Sun L., Lin L., Liu Z. (2021). Toward the commercialization of chemical vapor deposition graphene films. Appl. Phys. Rev..

[6] Li X., Wang R., Zuo Z., Ge L., Chen X., Xie X., Xiao L., Peng Y., Xu X., Hu X. (2021). Correlation between the response performance of epitaxial graphene/SiC UV-photodetectors and the number of carriers in graphene. Carbon.

[7] Sokolov D.A., Shepperd K.R., Orlando T.M. (2010). Formation of graphene features from direct laser-induced reduction of graphite oxide. J. Phys. Chem. Lett..

[8] Lin J., Peng Z., Liu Y., Ruiz-Zepeda F., Ye R., Samuel E.L., Yacaman M.J., Yakobson B.I., Tour J.M. (2014). Laser-induced porous graphene films from commercial polymers. Nat. Commun..

[9] Xu Y., Fei Q., Page M., Zhao G., Ling Y., Chen D., Yan Z. (2021). Laser-induced graphene for bioelectronics and soft actuators. Nano Res..

[10] Zang X., Jian C., Zhu T., Fan Z., Wang W., Wei M., Li B., Diaz M.F., Ashby P., Lu Z. (2019). Laser-sculptured ultrathin transition metal carbide layers for energy storage and energy harvesting applications. Nat. Commun..

[11] Aslam S., Sagar R.U.R., Liu Y., Anwar T., Zhang L., Zhang M., Mahmood N., Qiu Y. (2019). Graphene decorated polymeric flexible materials for lightweight high areal energy lithium-ion batteries. Appl. Mater. Today.

[12] Chen X., Luo F., Yuan M., Xie D., Shen L., Zheng K., Wang Z., Li X., Tao L. (2019). A dual-functional graphene-based self-alarm health-monitoring e-skin. Adv. Funct. Mater..

[13] Ye R., Chyan Y., Zhang J., Li Y., Han X., Kittrell C., Tour J.M. (2017). Laser-induced graphene formation on wood. Adv. Mater..

[14] Lee Y., Low M.J., Yang D., Nam H.K., Le T.D., Lee S.E., Han H., Kim S., Vu Q.H., Yoo H. (2023). Ultra-thin light-weight laser-induced-graphene (LIG) diffractive optics. Light-Sci. Appl..

[15] Peng Y., Zhao W., Ni F., Yu W., Liu X. (2021). Forest-like laser-induced graphene film with ultrahigh solar energy utilization efficiency. ACS Nano.

[16] Li J.T., Stanford M.G., Chen W., Presutti S.E., Tour J.M. (2020). Laminated laser-induced graphene composites. ACS Nano.

[17] Dixit N., Singh S.P. (2022). Laser-induced graphene (LIG) as a smart and sustainable material to restrain pandemics and endemics: A perspective. ACS Omega.

[18] He Q., Wang J., Wang G., Hao X., Li A. (2023). Construction of a durable superhydrophobic flame-retardant coating on the PET fabrics. Mater. Design.

[19] Wang J., Zhang Y., He Q. (2023). Stretchable superhydrophobic fluororubber fabricated by transferring mesh microstructures. Soft Matter.

[20] He Q., Ma Y., Wang X., Jia Y., Li K., Li A. (2023). Superhydrophobic flexible silicone rubber with stable performance, anti-icing, and multilevel rough structure. ACS Appl. Polym. Mater..

[21] Li A., Wei Z., Zhang F., He Q. (2023). A high reliability super hydrophobic silicone rubber. Colloid Surf. A.

[22] Huang L., Gu M., Wang Z., Tang T., Zhu Z., Yuan Y., Wang D., Shen C., Tang B., Ye R. (2021). Highly efficient and rapid inactivation of coronavirus on non-metal hydrophobic laser-induced graphene in mild conditions. Adv. Funct. Mater..

[23] Deshmukh S., Banerjee D., Quintero J.S.M., Fishlock S.J., Mclaughlin J., Waghmare P.R., Roy S.S. (2021). Polarity dependent electrowetting for directional transport of water through patterned superhydrophobic laser induced graphene fibers. Carbon.

[24] Farshchian B., Gatabi J.R., Bernick S.M., Lee G.H., Droopad R., Kim N. (2018). Scaling and mechanism of droplet array formation on a laser-ablated superhydrophobic grid. Colloid Surf. A.

[25] Chen B., Johnson Z.T., Sanborn D., Hjort R.G., Garland N.T., Soares R.R.A., Belle B.V., Jared N., Li J.Z., Jing D. (2021). Tuning the structure, conductivity, and wettability of laser-induced graphene for multiplexed open microfluidic environmental biosensing and energy storage devices. ACS Nano.

[26] Tiliakos A., Ceaus C., Iordache S.M., Vasile E., Stamatin I. (2016). Morphic transitions of nanocarbons via laser pyrolysis of polyimide films. J. Anal. Appl. Pyrol..

[27] Ye R., James D.K., Tour J.M. (2018). Laser-induced graphene. Acc. Chem. Res..

[28] Li S., Zhong M., Zou Y., Xu M., Liu X., Xing X., Zhang H., Jiang Y., Qiu C., Qin W. (2023). Fabrication of micron-structured heatable graphene hydrophobic surfaces for deicing and anti-icing by laser direct writing. Coatings.

[29] Wang Y., Wang Y., Zhang P., Liu F., Luo S. (2018). Laser-induced freestanding graphene papers: A new route of scalable fabrication with tunable morphologies and properties for multifunctional devices and structures. Small.

[30] Gupta A., Holoidovsky L., Thamaraiselvan C., Thakur A., Singh S., Meijler M., Arnusch C. (2019). Silver-doped laser-induced graphene for potent surface antibacterial activity and anti-biofilm action. Chem. Commun..

[31] Nasser J., Lin J., Zhang L., Sodano H.A. (2020). Laser induced graphene printing of spatially controlled super-hydrophobic/hydrophilic surfaces. Carbon.

[32] Han Y., Han Y., Sun J., Liu H., Luo X., Zhang Y., Han L. (2022). Controllable nanoparticle aggregation through a superhydrophobic laser-Induced graphene dynamic system for surface-enhanced Raman scattering detection. ACS Appl. Mater. Interfaces.

[33] Yang L., Shen X., Yang Q., Liu J., Wu W., Li D., Du J., Zhang B., Fan S. (2021). Fabrication of biomimetic anisotropic super-hydrophobic surface with rice leaf-like structures by femtosecond laser. Opt. Mater..

[34] Wang W., Lu L., Li Z., Xie Y. (2022). Laser induced 3D porous graphene dots: Bottom-up growth mechanism, multi-physics coupling effect and surface wettability. Appl. Surf. Sci..

[35] Ruan X., Wang R., Luo J., Yao Y., Liu T. (2018). Experimental and modeling study of CO_2_ laser writing induced polyimide carbonization process. Mater. Design.

[36] Wang W., Lu L., Xie Y., Wu W., Liang R., Li Z., Tang Y. (2021). Controlling the laser induction and cutting process on polyimide films for kirigami-inspired supercapacitor applications. Sci. China Technol. Sci..

[37] Tamrin K.F., Moghadasi K., Sheikh N.A. (2020). Experimental and numerical investigation on multi-pass laser cutting of natural fibre composite. Int. J. Adv. Manuf. Technol..

[38] Venkatachalam S., Depriester M., Sahraoui A.H., Capoen B., Ammar M.R., Hourlier D. (2017). Thermal conductivity of Kapton-derived carbon. Carbon.

[39] Li Z., Deng L., Kinloch I.A., Young R. (2023). Raman spectroscopy of carbon materials and their composites: Graphene, nanotubes and fibres. Prog. Mater. Sci..

